# Active Decision Support System for Observation Scheduling Based on Image Analysis at the BOROWIEC SLR Station

**DOI:** 10.3390/s22208040

**Published:** 2022-10-21

**Authors:** Tomasz Suchodolski

**Affiliations:** Centrum Badań Kosmicznych Polskiej Akademii Nauk (CBK PAN), Bartycka 18A, 00-716 Warszawa, Poland; suchodolski@cbk.poznan.pl

**Keywords:** satellite laser ranging (SLR), space debris, passive optical acquisition, active scheduling system, trajectory tracking, predictive control

## Abstract

The dynamic exploration of the orbits from the LEO-to-GEO region, for the needs of telecommunication services, science, industry and defense, forces monitoring of the trajectory of such orbital objects for the safety of spacecraft traffic and, in the case of deorbitation, for the safety of ground infrastructure. First off all, the need for trajectory monitoring in order to avoid collisions can be distinguished, as well as the need to calibrate the satellite on-board devices. This is mainly carried out by radar measurements, by passive optical acquisition and active laser measurements. The number of orbital objects increases rapidly, and the number of tracking stations for the second is relatively small. This leads to a situation in which each tracking station must select which of the objects will be subject to the measurement task. In the case of the Satellite Laser Ranging (SLR) or passive optical set-up, the weather conditions are an important factor enabling the measurement of the orbital object trajectory. This paper presents an innovative observation scheduling support system based on the analysis of the images obtained from the Allsky camera. The information of the degree of cloud cover, the position of the Sun/Moon in connection with the graphical projections of the ephemeris trajectory of the orbital objects allows increasing the measurement efficiency. The presented solution is part of a larger number of improvements carried out by the author, which lead to the upgrade of SLR stations in terms of new technologies and safety of use.

## 1. Introduction

The issue of laser ranging to artificial satellites dates back to 1964, when the first measurement of the distance between the SLR station and the Beacon-B Explorer satellite was made [1]. Historically, laser stations were used to measure the trajectories of geodynamic satellites [2,3], scientific platforms and robotic rovers located on the surface of the Moon [4,5], as well as to determine the position of the first navigation constellations [6,7]. As technology advances, laser measurements are used to calibrate altimetric satellites, the correct operation of which depends on accurate information about their location [8,9]. SLR measurements make a contribution to research on the implementation and definition of the International Terrestrial Reference Frame (ITRF), pole movement, the coordinates of the geocenter of the Earth’s gravitational field, the coordinate and velocity changes in the position of the SLR stations and determining the position of objects orbiting Earth with centimeter accuracy [10,11]. The greatest advantage of the laser technique is the direct and absolute measurement of the range between the SLR observatory and the given orbital object (satellite, rocket bodies and space debris).

Over the years, more and more satellites being launched into orbit have led to the situation that, in space, there is more space debris than active operating satellites. According to the European Space Agency Space Debris Office (ESA SDO) report [12], there is a sharp increase in the number of orbital objects, resulting in an avalanche of debris objects accompanying the main space mission. Among them, we can distinguish rocket bodies, payload fragmentation debris and rocket fragmentation debris (Table 1). Although these are irrelevant elements from the point of view of the orbital mission, they pose a high risk of possible collisions. An important factor is that such elements are uncontrollable, because they do not have mechanisms that would allow for changing the orbit trajectory. Therefore, in the event of a collision probability, it is not possible to correct their orbit. Moreover, not all operational satellites have the possibility to correct their trajectory. It should be mentioned that small or inactive satellites do not have such maneuvering mechanisms. This makes it necessary to also track such objects to prevent possible collisions. In connection with the above, there is also a need to determine the trajectory for objects such as space debris or defunct satellites.

Unfortunately, accurate information about the trajectory of an orbital object is not always sufficient. In the case of rocket bodies, information about the orientation of such elements is also important. It may turn out that knowing the exact trajectory of the rocket segment is insufficient, since the uncertainty of its orientation may lead to a collision. The difference between the diameter of a rocket segment (e.g., 3 m) and its length (e.g., 8 m) and its specific orientation in space (along or perpendicular) may result in erroneous assumptions for the collision probability calculations. Another important effect is the possibility of object rotation. The rotation effect can lead to the fragmentation of the orbital object and the creation of another pool of debris objects. All these uncertainties can be reduced by laser ranging to an orbital object. Short laser pulses (ps to ns) enable accurate measurement of the object’s position, and with in-depth data analysis they also provide information about the object’s orientation [13] or its rotation (spin) [14]. Therefore, the use of laser technology in recent years has changed the pool of objects that need to be tracked.

Most of the existing SLR stations support only scientific missions and navigation constellations. Of the 40 operational stations, only a small number are able to successfully track objects such as space debris. Due to the increasing number of scientific missions, the list of objects for laser tracking exceeds 100 objects per station. In the case of low Earth orbit (LEO) objects, multiple passes of a satellite over a station often happens, so it increases the observation load of the given station. Therefore, the method of the observations planning process is important in order to maximize the operational capacity of the SLR station. As long as it is possible to perform a priori ephemeris analysis for passes over the given SLR station, the weather conditions for the next few hours are not known at this stage. It often happens that partial cloud cover makes it impossible to observe a given object. It follows that it is necessary to introduce such a functionality of the planning system so that it takes into account the current weather conditions. This paper presents an innovative decision support module in the SLR observation planning system based on the postprocessing and analysis of the images taken from a fish-eye camera and available ephemeris of orbital objects. This solution is a fragment of a series of improvements made by the author described partially in the work in [15].

## 2. Satellite Laser Ranging Process

**Figure 1:**
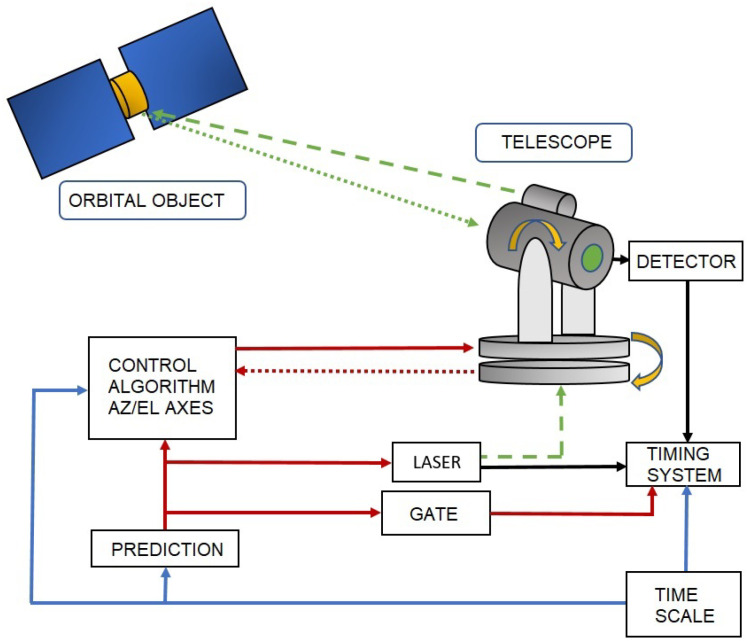
Block diagram of the SLR tracking system.

Laser ranging to an orbital object is carried out by using a laser source with short pulse width (in units of ps or of ns level) and specified repetition rate (in units of Hz or of kHz level). The laser beam is transmitted towards the theoretical orbital position of the object (κ) in order to obtain the signal echoes ($E_{x}\left(t\right)$). The time interval $\Delta t_{p}$ between the moments of time of sending the laser pulse ($t_{START}$) and recording the photon event ($t_{STOP}$) scattered from the object in the telescope’s photon detector determines the double distance to the object in the time domain (Figure 1).

Laser measurements are made for satellites, rocket bodies and space debris from the regions from LEO (low Earth orbit) to GEO (geostationary orbit), so the distance between the ground station and the orbital object ranges mainly from about 350 km to 42,000 km. The tracked objects differ in terms of their purpose, so they also differ in the shape and materials from which they are made. This determines the method of their observation and the selection of laser sources to obtain effective results.

### 2.1. Types and Characteristics of Tracked Orbital Objects

The orbital objects tracked by SLR stations can be broken down into two main categories: cooperative and uncooperative targets. Cooperative targets, mainly active satellites, are equipped with systems of cubic prisms (retroreflectors) mounted on the outer casing, whose task is the low-loss reflection of the laser beam emitted from the SLR station. Uncooperative targets are therefore all other objects that are not equipped with cubic prism (satellites, rocket bodies and space debris) or decommissioned satellites equipped with retroreflectors with lost ability to maintain spatial orientation (wrong orientation makes it impossible to reflect the beam towards the station). While in the case of ranging to the cooperative targets in connection to mounted retroreflectors there is no need to generate highly energetic laser beams, in the case of uncooperative objects, there is a need to use high-energy laser sources to obtain scattered echoes from the orbital object [16,17,18]. This means that the observation of such different objects requires the use of two different laser sources. It is also dictated by the safety of instruments located on scientific satellites (e.g., Earth observation). In a simple approximation, high-energy lasers emit radiation to inactive targets. The use of two different laser sources for the observation of two different types of objects must therefore be taken into account in the observation planning process.

**Figure 2:**
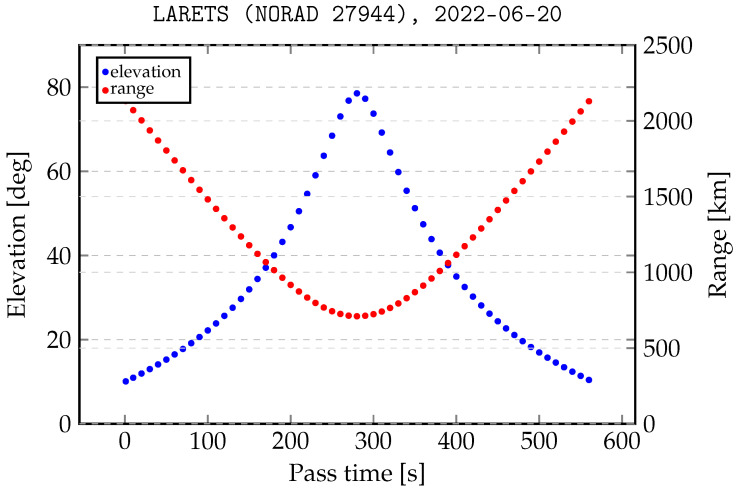
Elevation angle and distance to the LARETS satellite in relation to the SLR station.

Another key element in the observation planning process is the difference in the reflection characteristics of cooperative and uncooperative targets with an increase in the distance depending on the type of orbit. The Borowiec SLR station (LASBOR) tracks cooperative and uncooperative targets from the LEO regime and cooperative targets up to the MEO region (medium Earth orbit). For example, Figure 2 shows the pass over LASBOR station of a typical cooperative object (LARETS geodetic satellite) from the LEO region. As can be seen, the range varies from 710 km to 2130 km depending on the angle of pass elevation.

**Figure 3:**
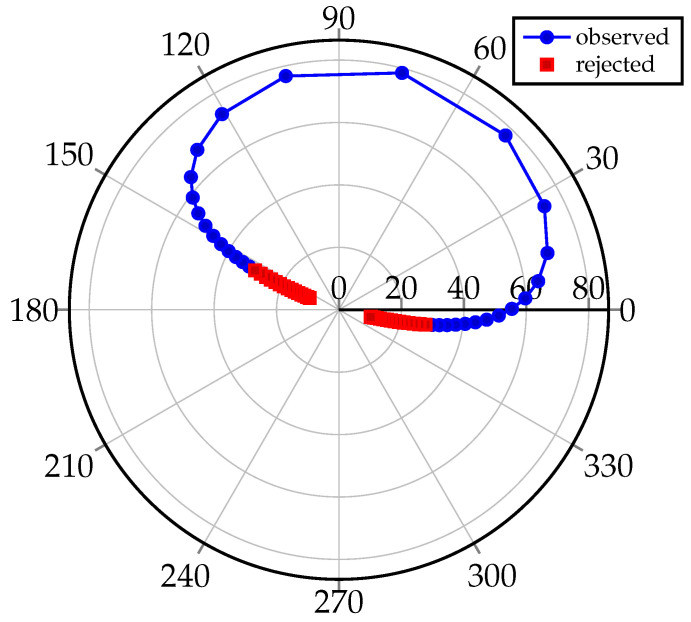
Elevation and azimuth angles to the LARETS satellite in relation to the SLR station.

In the case of objects equipped with retroreflectors, they do not cause perturbations in the ranging process to the orbital object in terms of laser pulse energy level. However, in the case of uncooperative objects as rocket bodies, such a change in distance may make it impossible to carry out the measurement process due to the lack of signal echoes. For the LASBOR station, the range limit to uncooperative targets results is 1000 km for RCS=10 (Radar Cross-Section). Effects related to light refraction are another issue, but they are not the subject of this paper. When analyzing the above limits, it can be concluded that it is necessary to take into account these limitations in the process of observation scheduling (Figure 3).

On the basis of subsequent measurements, the limits of the elevation angles were established. For the LEO region, for cooperative objects, it is 20 degrees above the horizon, for uncooperative targets, 30 degrees, respectively.

**Figure 4:**
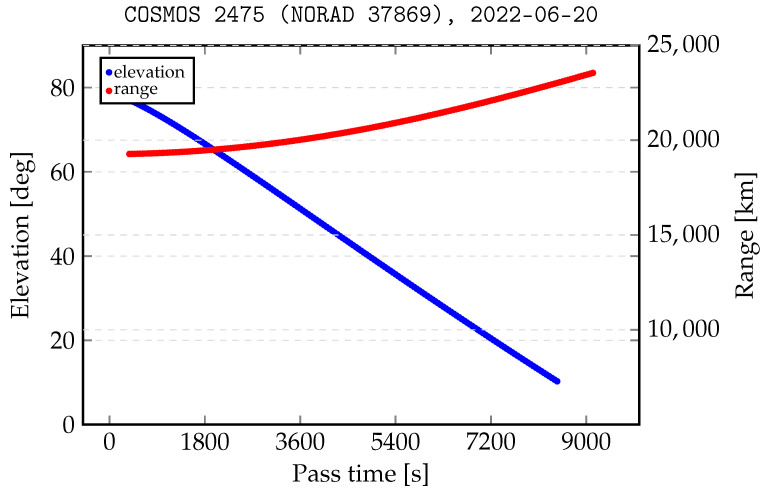
Elevation angle and distance to the COSMOS 2475 satellite in relation to the SLR station.

Laser ranging to MEO targets has a different specificity. The pass of the orbital object over the station takes a relatively long time (tens of minutes to hours). Therefore, unlike ranging to LEO objects, whose passes last for a few minutes, observations of MEO objects can be split into several slots overnight. Another aspect is that for the MEO region, ranging to cooperative targets is only possible. Figure 4 shows the change in the distance and elevation angle of the COSMOS 2475 tracking process. Due to the high orbital altitude, the distance to the object changes less as a percentage than it does for LEO objects (19,276 km–23,553 km).

**Figure 5:**
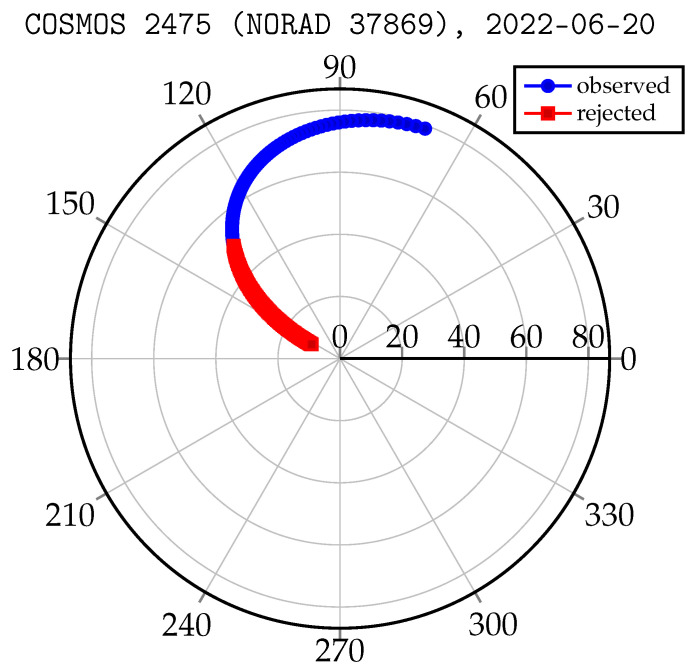
Elevation and azimuth angles to the COSMOS 2475 satellite in relation to the SLR station.

An important aspect is that the low elevation observation angle of the orbital object implies a decrease in measurement efficiency due to the location (nadir) of the retreflectors’ panel and their acceptance cone. Taking into account the above facts, the process of scheduling the observation of the MEO objects can be limited to passes starting from 50 degrees above the horizon. The advantage of such a solution is the release of observation slots for relatively short observations of objects from the LEO region, as shown in the Figure 5.

Determining the boundary conditions during the observation planning process increases the number of observation slots, taking into account the orbital region (LEO–MEO) and the type of the tracked object (cooperative or uncooperative). Such a priori scheduling enables an orderly process of observation of orbital objects. The only unknown variable is the weather conditions that will occur when the observations are made. The full cloud cover or rainfall make the measuring process completely impossible, while in the case of partial cloud cover, it is possible to select objects such that it is possible to perform object tracking.

### 2.2. Weather Conditions and Operational Time

**Figure 6:**
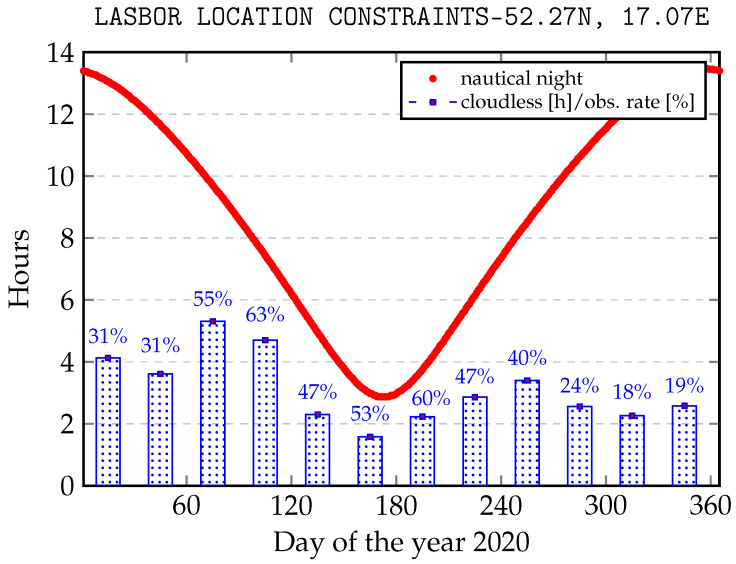
Effectiveness of successful observations depending on the season.

In the present configuration, the SLR LASBOR tracks orbital objects within the nautical night. This implies a variability in the number of daily observing hours over the year. Weather conditions, which strongly depend on the season in Europe, are another aspect. Unfortunately, during the longest nautical nights rainfall and full cloud cover make it difficult to track objects effectively. Figure 6 shows the length of the nautical night over the year and the percentage of weather conditions suitable for tracking operation.

As shown, the winter season is characterized by a high degree of cloud cover, which implies a relatively low number of observation hours at night time (e.g., in January 4,13 h of cloudless of the statistical night constitutes 31% of observation rate). The months from March to September are characterized by the best weather conditions and observation results despite the shorter nights. In connection with the above, there is a need to optimize the planning process in the remaining months. It often happens that only part of the sky is covered with the clouds. Additionally, this fact should be resolved when real-time observations are being made.

### 2.3. Observation Load

Currently, the list of objects tracked by SLR LASBOR station has over 200 orbital objects. It can be assumed that half of the objects are cooperative targets (LEO–MEO); the rest are rocket bodies from the LEO region. This replicates the generation of extensive lists of scheduled measurements. For example, for the night of 2021/12/15, the scheduled observation list included 249 passes. As shown in Table 2, the scheduled number of 200 tracked objects from different orbital regions causes overlaps. The situation takes place despite the introduction of boundary conditions for the observation of a given type of object and given orbital altitude, as described in the Section 2.1.

Therefore, selecting a specific object for the tracking process is difficult issue [19]. Initially, the selection may be based on the time of the pass over the station and knowledge of the object’s reflection characteristics, as well as the time elapsed since the last measurement. At this stage, the operator of the SLR system has no knowledge of the weather conditions described in the Section 2.2.

## 3. Active Decision Support System for Observation Scheduling and Observation Safety

Appropriate planning of the observations (Section 2.3), taking into account the specificity of orbital objects (Section 2.1), enables the operator to select objects and time slots that generate the highest measurement efficiency in terms of the time length of the pass over the SLR station (Table 2). The only unknown for the system operator is the current weather conditions (Section 2.2). For night time operations, the use of a simple Allsky camera in connection with a low light pollution index at the station location does not bring any effect (e.g., illumination of cloud fractions). Short exposure times do not show fractions of clouds or stars with low brightness. Too long exposure times introduce noise and highlight bright stars despite light cloud cover. Regardless of the above, the operator’s analysis of such an image in an illuminated room is a demanding task that requires attention. This condition forced the development of such an image postprocessing system that the operator could clearly and easily assess the weather conditions above the station. Additionally, the existing ephemeris of the objects can be projected onto the image acquired from the Allsky camera in the form of objects trajectories.

### 3.1. Data Structures

**Figure 7:**
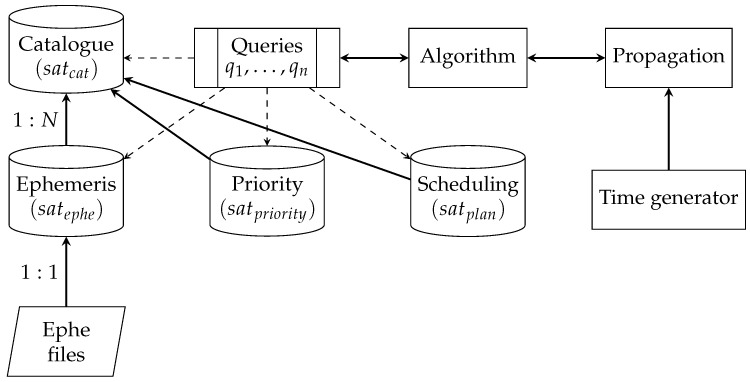
Block diagram of the database structure—scheduling chain.

Due to the large number of tracked objects, the catalog of the objects ($sat_{cat}$), ephemeris information ($sat_{ephe}$), the acquired measurement data ($sat_{meas}$) and other derived data ($sat_{x}$) necessary to carry out the process are stored in the SQL database environment, the processing of which is thus ordered and is carried out in accordance with the following scheme:

(1) $$\{\left(sat_{cat},sat_{ephe},sat_{plan},sat_{meas},\ldots ,sat_{n}\right)|q_{1},\ldots ,q_{k}\},$$

where: $sat_{*}$ are attribute relationships, and $q_{1},\ldots ,q_{k}$ are a relational algebra of the laser management system. This method of data organization, a fragment of which is shown in the Figure 7, also allows for the subsequent analysis of the collected information, which can be used, for example, to generate parameters of new orbits or to characterize given objects.

**Figure 8:**
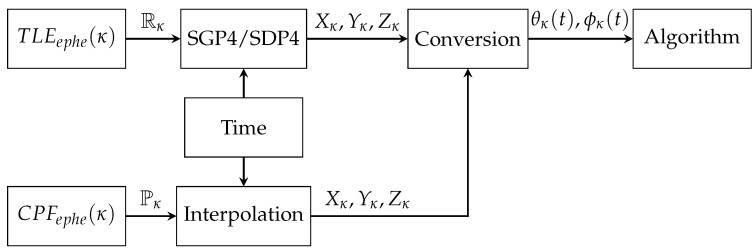
Block diagram of the ephemeris processing data flow.

The process of tracking an orbital object $\kappa \in sat_{cat}$ in the time domain (t) requires the conversion of ephemeris information $sat_{ephe}\left(\kappa \right)$ about the theoretical spatial position $X_{\kappa },Y_{\kappa },Z_{\kappa }$ of the orbital object into the values of azimuth $\theta_{\kappa }\left(t\right)$ and elevation $\phi_{\kappa }\left(t\right)$ angles that enable the follow-up movement of the telescope mount behind this object (Figure 8).

(2) $$sat_{ephe}\left(\kappa \right)\Rightarrow X_{\kappa }\left(t\right),Y_{\kappa }\left(t\right),Z_{\kappa }\left(t\right)\to \theta_{\kappa }\left(t\right),\phi_{\kappa }\left(t\right).$$

The role of the management software is, inter alia, processing information about the theoretical position of the orbital object $\kappa \in sat_{cat}$ in the time domain (t) into control data vectors $V_{Az/El}=\left[v,a,\theta ,\phi ,\ldots \right]$ of drives of the Az/El orthogonal axis of the telescope mount and controlling this process. By obtaining information about the azimuth and elevation angles in the time domain, it is possible to visualize the object’s trajectory by comparing these data with weather data.

### 3.2. Image Processing

The assumption was to use publicly available software for the analysis and processing of optical data obtained from the Allsky camera. While the entire station’s management software was created in C# in conjunction with MySQL, the external support software uses different environments. For data processing in the form of images obtained from the Allsky camera, it was decided to use OpenCV using Python scripts. The OpenCV library provides extensive functions that enable image processing that meet the needs of this solution. The proposed implementation does not require real-time processing, but the available functions shortened the time of creating the active decision support system.

**Figure 9:**
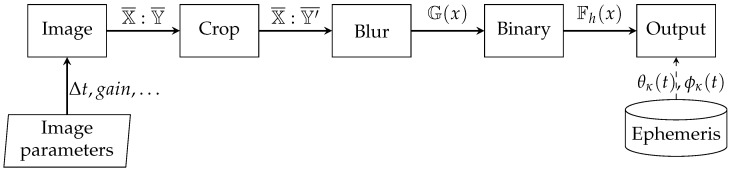
Block diagram of the day time image processing.

The aim of the task is to determine the degree of cloud cover during the day and night time. Therefore, a different course of action has to be implemented during the day time with the sunlight and a different one during the night time, which is strictly dependent on the light pollution. Simple operations were chosen to construct the image processing algorithm, which in changing weather conditions simplifies the calibration and modification of the actions taken. For operations carried out during the day, the algorithm was implemented according to Figure 9. Due to the fact that most of the available cameras have detectors with a different number of pixels in the X:Y axes (e.g., 1944 px:2592 px), the first operation is to trim these axes to equal values ($\bar{X}:\bar{Y^{\prime }}\in \left\{\bar{X}\wedge \bar{Y}\right\}$). In order to remove high-frequency content in the form of noise or edges, Gaussian blurring G(x) was used. The purpose of all day time operation is not to detect shapes but only changes in background brightness. The image prepared in this way can be binarized by specifying the activation threshold $F_{h}\left(x\right)$. Therefore, the image was divided into areas with two degrees of brightness, understood as overcast or lack thereof. Finally, as the upper graphical layer, ephemeris data are added in the form of the satellite pass trajectory built on azimuth $\theta_{\kappa }\left(t\right)$ and elevation $\phi_{\kappa }\left(t\right)$ angles. By comparing the binarized image with the pass trajectory, it is possible to estimate the percentage of satellite visibility in order to perform a laser measurement. Taking into account all satellites passes at the given moment of time (e.g., Table 2), it is possible to choose the one pass with the longest visibility.

**Figure 10:**
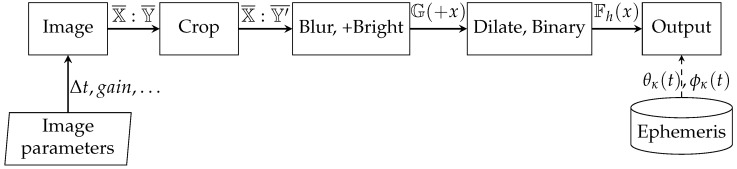
Block diagram of the night time image processing.

**Figure 11:**
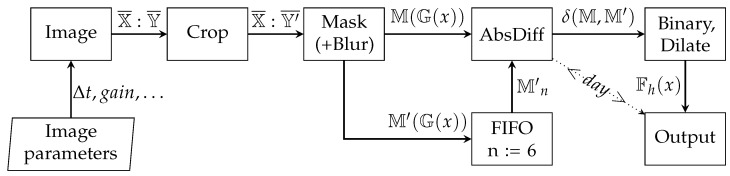
Block diagram of the cumulative differential image processing.

The night time processing mode (Figure 10) differs in the way the data are handled. Due to the possible different cloud bases (mainly dependent on the season), and therefore a change in the background constant brightness (light pollution), the night time image analysis is best performed by star detection. The second approach is the differential analysis of consecutive images (Figure 11). Even in the case of full cloud cover, it is possible for the system operator to observe the background changes and estimate the overcast level depending on the wind (cumulative differential image changes).

## 4. Experiment

### 4.1. Test Platform

The assumption of the project was to use cheap and generally available elements in the form of a camera and the Raspberry Pi microcontroller. Initially, a camera dedicated to Raspberry Pi with the resolution of 1944 px:2592 px was used. Unfortunately, the small pixel size of the camera prevents correct acquisition during night time by applying long exposures that resulted in noise. Therefore, the astronomical ASI120 monochrome camera [20] (960 px:1280 px) with the pixel size of 3.75 μm was selected. The result of the postprocessing in the form of images was made available on the Apache website to be accessible from any SLR station computer.

### 4.2. Active Decision Support System Operational Test

**Figure 12:**
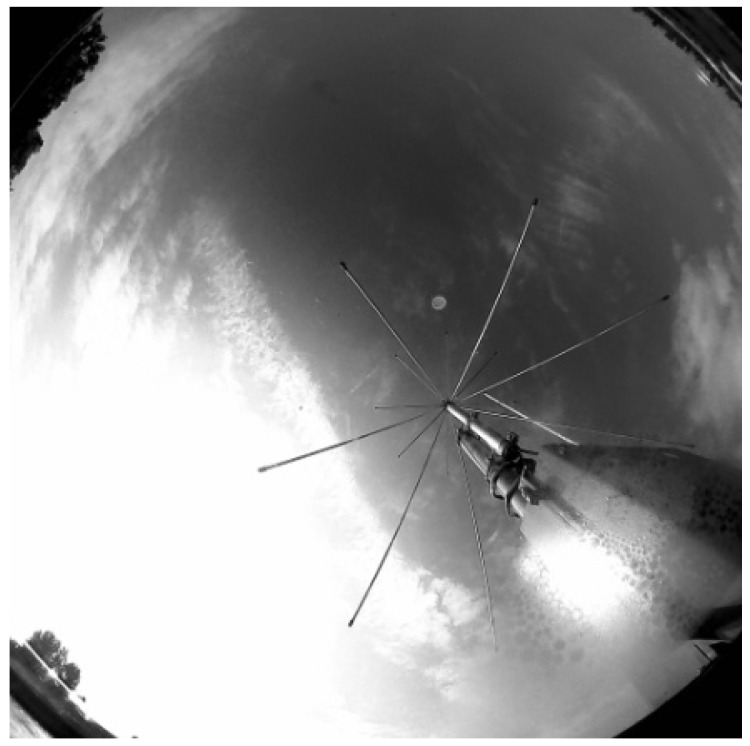
Cropped unprocessed image.

**Figure 13:**
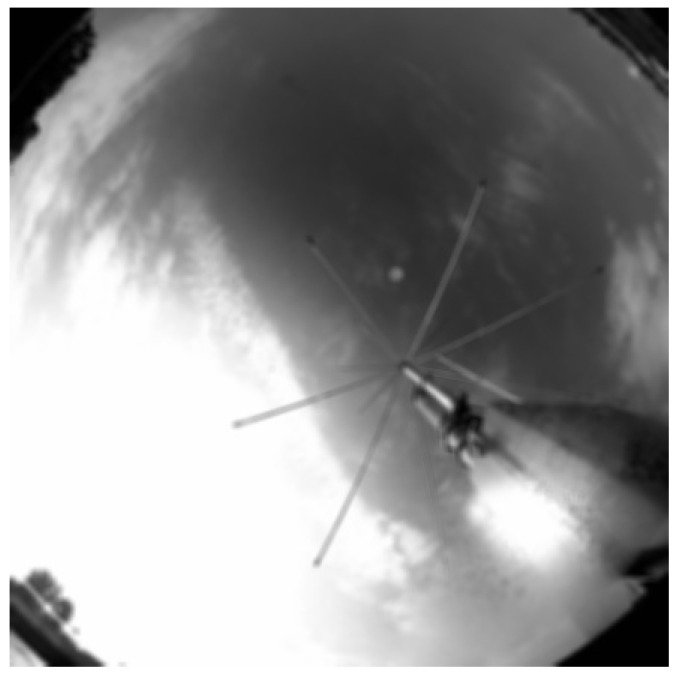
Blurred image.

**Figure 14:**
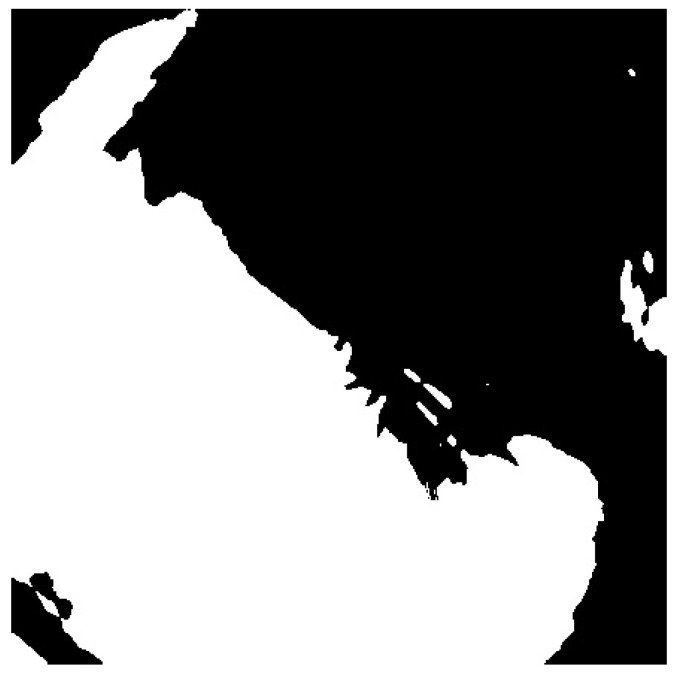
Binary transformation.

**Figure 15:**
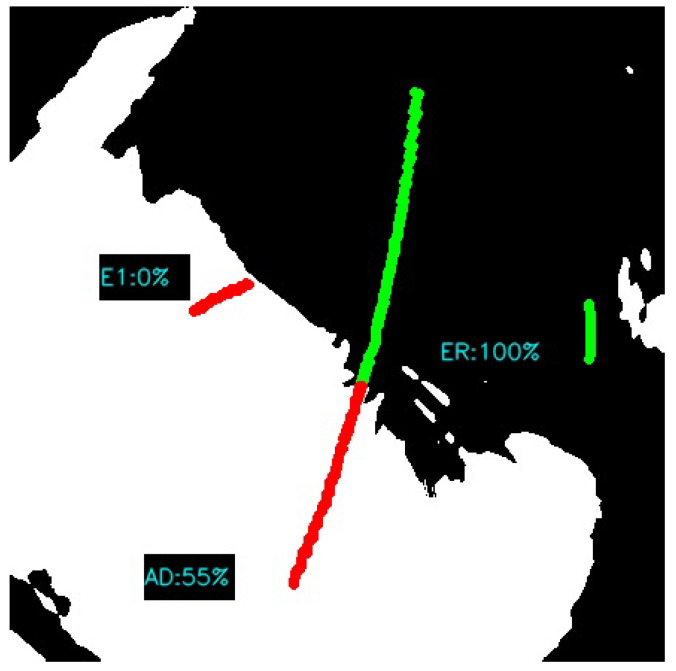
Ephemeris graphical projection (green line—cloudless, red line—overcast).

Functional tests were carried out in day and night mode. In accordance with the scheme of operation shown in Figure 9, successive transformations of the image were performed in the day time (Figure 12, Figure 13, Figure 14 and Figure 15). It should be noted that the calibration frames known from astronomy in the form of flat, bias or dark frames were not used.

For that moment of time, there were 3 objects from the tracking list (Etalon-1, ERS-1, ADEOS-1) within the measurement range of the SLR station. As shown in Figure 15, the percentage value of visibility for each object in relation to the current cloud cover was estimated (where green markings mean favorable conditions). These values were, respectively: Etalon-1 = 0%, ERS-1 = 100% and ADEOS-1 = 55%. The validity of the estimate depends on the predicted duration of the satellite’s pass in relation to the speed of cloud movement and is only illustrative for the given moment of time.

**Figure 16:**
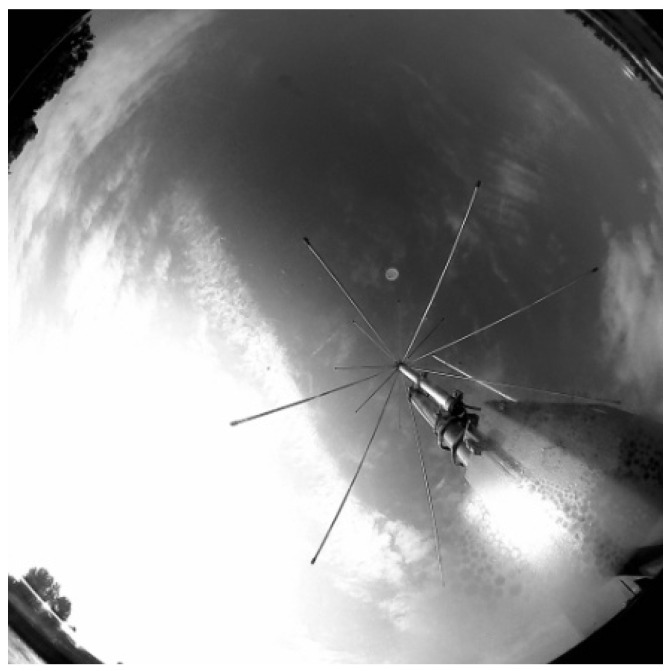
Cropped unprocessed image.

**Figure 17:**
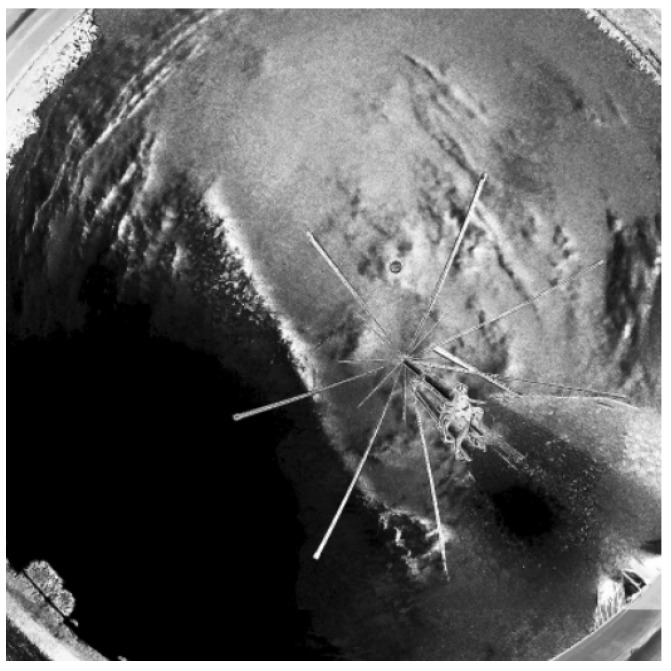
Cumulative differential image.

Additionally, as a supplementary task, it was decided to perform tests of the cumulative differential image algorithm (Figure 11), the result of which is presented in the confrontation of the original image in Figure 16 and Figure 17. This method of processing revealed cloud fractions seemingly invisible to the human eye.

**Figure 18:**
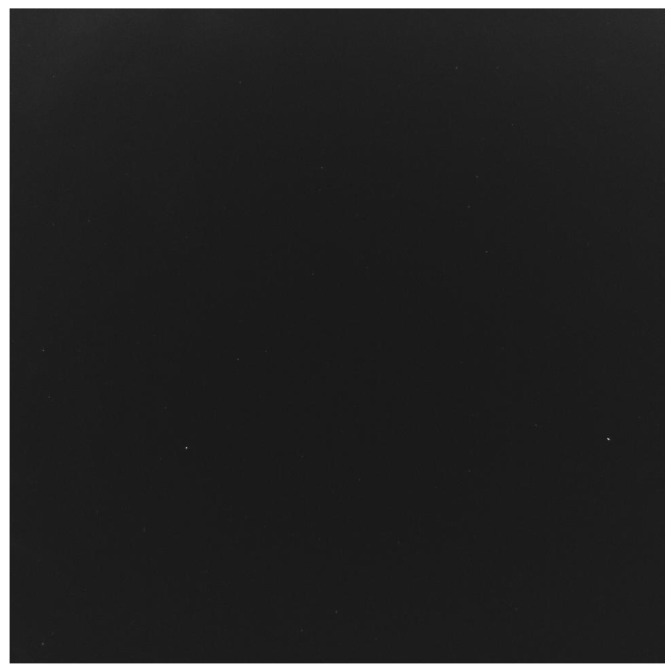
Cropped unprocessed night image.

**Figure 19:**
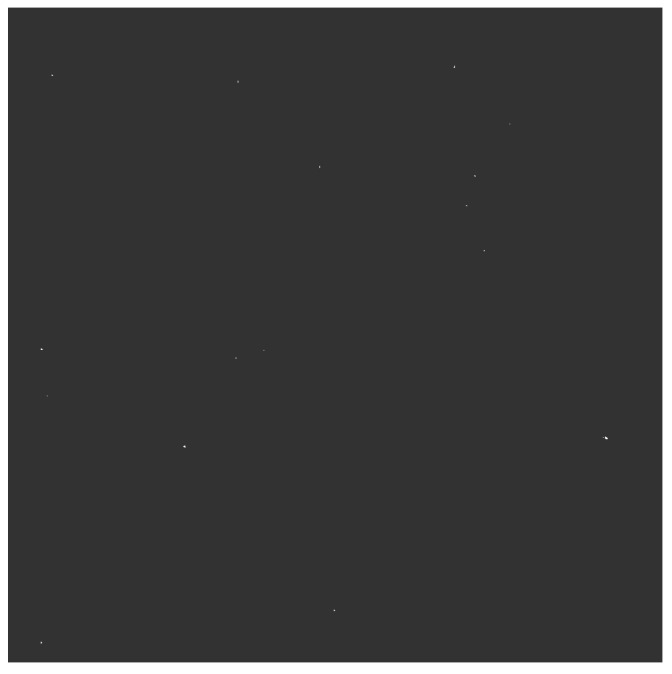
Blurred and brightened image.

**Figure 20:**
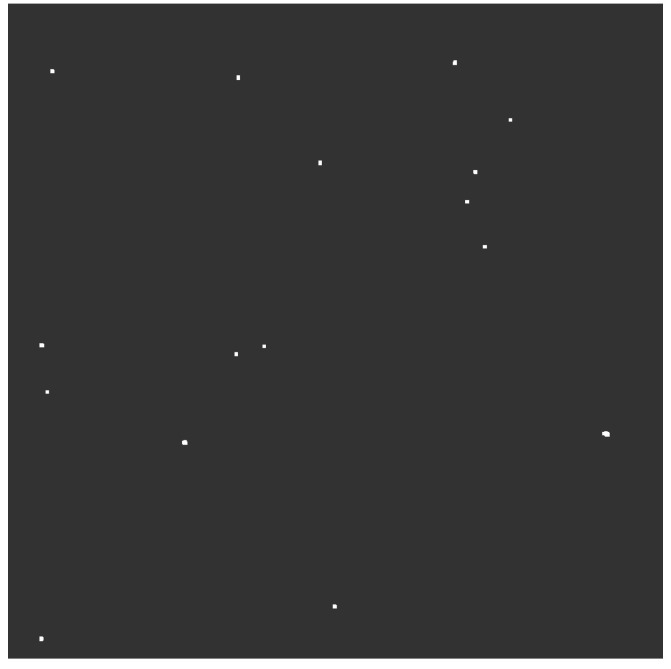
Dilation transformation.

The estimation of the degree of cloud cover in the night mode differs significantly. As described in Section 3.2, the best unequivocal method is star detection. In order to minimize image noise, which can lead to misidentification of the stars, relatively short exposure times were used. This method of acquisition makes it difficult to perform analysis by a human but is not a problem for processing algorithms. Only two brighter stars can be seen on the unprocessed and cropped image shown in the Figure 18, and it would require an operator’s effort to analyze such dark graphic. In accordance with the scheme of operation shown in Figure 10, after the blur operation, the image was brightened (Figure 19), and the stars were highlighted using the dilate process (Figure 20). At this point, the number of visible stars rises to 16 and they are easily seen. The last operation on the image marks the cloudless areas in the form of circles and makes the graphic projection of the trajectories of the object’s passes over the SLR station.

**Figure 21:**
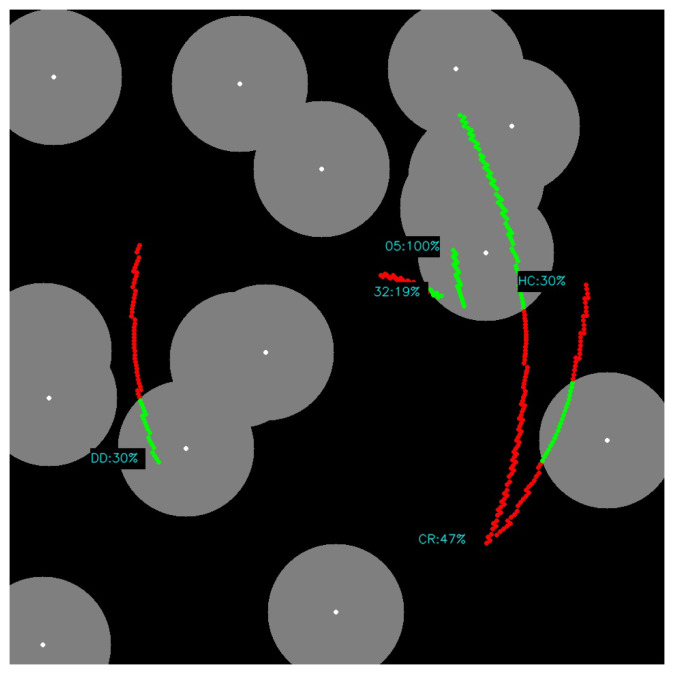
Ephemeris graphical projection (green line—cloudless, red line—overcast).

For that moment of time, there were 5 objects from the tracking list (rocked body SL3–13121, satellittes: GLONASS 105, GLONASS 132, Cryosat–2 and HY–2C). As shown in Figure 21, the percentage value of visibility for each object in relation to the current cloud cover was estimated (where green markings means favorable conditions). These values were, respectively: SL3-13121 = 30%, GLONASS 105 = 100%, GLONASS 132 = 19%, Cryosat-2 = 47% and HY-2C = 30%. The validity of the estimate depends on the predicted duration of the satellite’s pass in relation to the circles around stars with arbitrarily selected diameters and is only illustrative for the given moment of time. Field flattening (circles) was also omitted due to the lack of a target lens (ultimately 180${}^{\circ }$ field of view).

**Figure 22:**
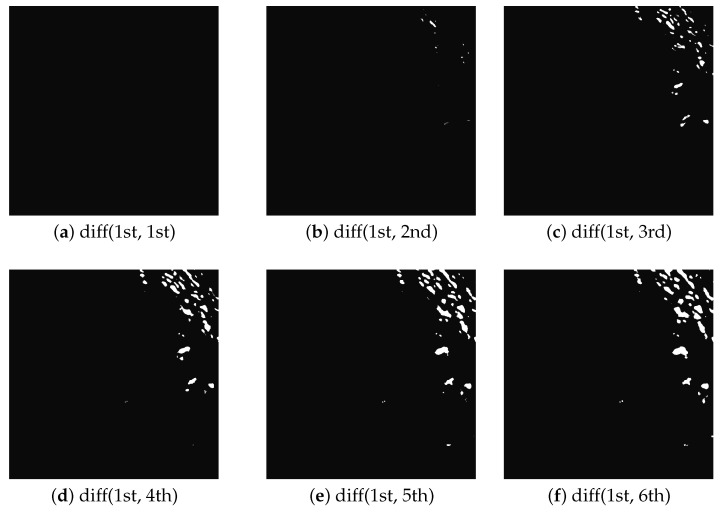
Cumulative differential night images (n:=6).

**Figure 23:**
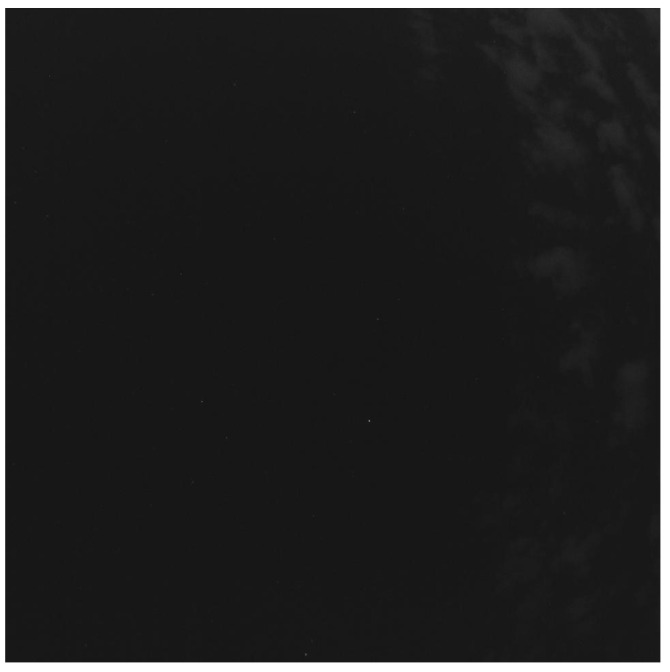
1st night frame (starting minute).

**Figure 24:**
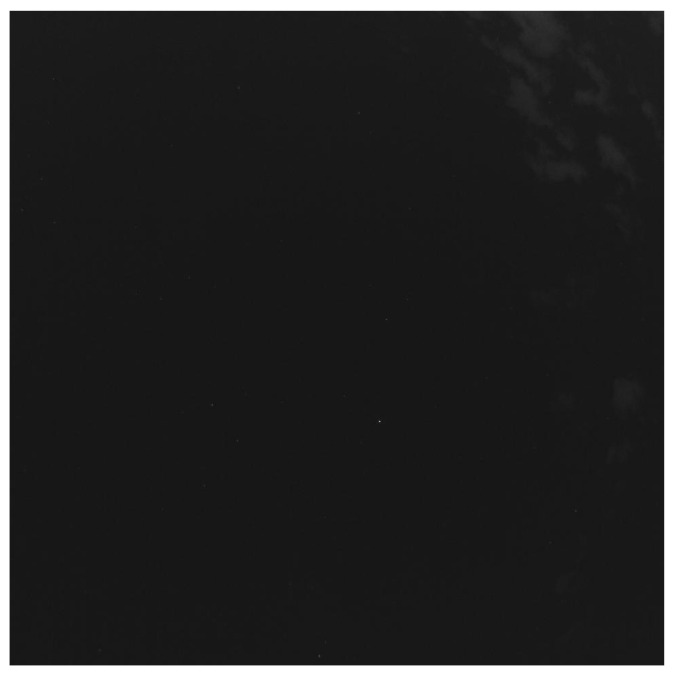
6th night frame (+6 min).

Additionally, a test of the cumulative differential image algorithm was performed for the night mode, the steps of which are shown in the Figure 22. Figure 23 and Figure 24 show two night images with the cloud fraction taken 6 min apart. As can be seen, cloud fractions are subtle. From the point of view of the system operator, it is not possible to state changes in cloud cover. At the moment, the station operates only in night mode, so it is impossible to analyze such images in a lit room.

The basis of the algorithm (Figure 11) is image acquisition in every minute and cumulative differential comparison every *n* minutes (in tests n:=6). Figure 22a–f shows the results of such differential comparisons. The self-differential comparison (a) suppresses the details, which confirms the correct operation of the algorithm. With each successive image (b–f) compared with the base image (1st one), the differences are emphasized. After n:=6 images is executed, the new base image gets into the FIFO buffer as a reference one. If each captured image was analyzed separately (Figure 23 and Figure 24), it would be difficult or even impossible to determine the cloud cover level in a lit operator’s room. The algorithm shown in Figure 11 highlights any background changes that occur over time (Figure 22b–f), making these changes as white fractions. This way of presenting data does not require increased attention from the station personnel, who can focus on other tasks.

## 5. Conclusions

The process of laser ranging with the continuous growth of orbital objects and a limited number of the SLR stations is a complex issue. After decades of operational support for scientific missions, there is an additional need to monitor space debris objects. Despite the advanced observation planning algorithms, the weather factor is the main element decision uncertainty, which implies measurement efficiency. This paper proposed solutions to improve the effectiveness of measuring the distance to the orbital objects, taking into account weather conditions at the Borowiec SLR station. The presented algorithms for the active decision support system for observation scheduling led to an increase in the effectiveness of measuring positions of orbital objects. SLR Borowiec, so far, did not have the Allsky system, so the high effectiveness of observation was obtained only during cloudless nights. In other cases, the station operator had to manually check the condition of the cloud cover, or in the case of partial cloud cover, some measurements were ineffective. The application of the presented solution simultaneously provides information on weather conditions and enables the selection of such passes that generate maximum measurement efficiency. An additional advantage of this solution is the ability to support decisions in 24/7 operations, although the station currently only works in night mode. A separate issue is the parameterization of algorithms, which depends on the specific setup. Initially, the tests were carried out on a camera dedicated to Raspberry Pi, which enabled the analysis of ∼176${}^{\circ }$ of the sky area. Then, the camera was changed to an ASI120, with the lens enabling ∼120${}^{\circ }$ field detection. Therefore, the field flatness parameterization was abandoned partly until the target lens is used. Due to the changing weather conditions, depending on the season, the complete system parameterization will take many months. Variable background brightness dependent on light pollution will force the use of additional algorithms for changing the camera’s sensitivity. Additional improvements to the algorithms are possible, which may take into account air traffic using the ADS-B signal.

## Figures and Tables

**Table 1:** Evolution of numbers of orbital objects [12].

Year	Payload	Rocket Body	Payload Fragments Debris	Other Types ^1^
2021	7849	1990	7629	12,557
2020	6263	1957	6686	12,419
2015	3864	1965	6035	5789
2010	3268	1621	6191	4164
2005	2709	1391	1068	3595
2000	2493	1351	945	3438
1990	1708	1029	1030	2493
1980	938	584	547	2018
1970	402	209	196	1170

^1^ Others including: payload debris, rocket fragmentation debris, rocket mission related object and unidentified.

**Table 2:** SLR Borowiec observation list: 8 of 249 rows (2021/12/15—night tracking).

Object	Start [UTC]	Stop [UTC]	Pass [m:s]	Elevation [°]	Range [km]
GLN-134	16:07:50	16:37:51	30:01	50–75	19,328–19,518
Stella	16:07:50	16:13:25	05:35	20–65	881–1787
SL16-20625	16:10:38	16:14:19	03:41	30–38	1272–1473
SL8-11327	16:16:21	16:17:21	01:00	30–30	1685–1698
SL14-22784	16:18:52	16:23:29	04:37	30–42	1310–161
SL16-22220	16:19:25	16:24:52	05:27	30–58	987–1486
Galileo-211	16:27:37	16:57:38	30:01	50–60	23,921–24,433
CZ2C-28480	16:31:22	16:33:27	02:05	30–33	1174–1256

## Data Availability

Not applicable.
